# Herbal Medicines for Cold Hypersensitivity in the Hands and Feet: A Systematic Review and Meta-Analysis

**DOI:** 10.1089/acm.2018.0009

**Published:** 2018-12-14

**Authors:** Jun-Sang Yu, Dongnyung Lee, Daesung Hyun, Sei-Jin Chang

**Affiliations:** ^1^ Department of Sasang Constitutional Medicine, College of Korean Medicine, Sangji University, Wonju, Republic of Korea.; ^2^ Department of Gynecology Medicine, College of Korean Medicine, Semyung University, Chungju, Republic of Korea.; ^3^ Department of Preventive Medicine, The Graduate School of Yonsei University, Wonju, Republic of Korea.; ^4^ Department of Biostatistics and Computing, The Graduate School of Yonsei University, Wonju, Republic of Korea.; ^5^ Department of Preventive Medicine, Wonju College of Medicine, Yonsei University, Wonju, Republic of Korea.; ^6^ Institute of Environmental & Occupational Medicine, Wonju College of Medicine, Yonsei University, Wonju, Republic of Korea.

**Keywords:** cold hypersensitivity, coldness, Raynaud's phenomenon, herbal medicine, systematic review, meta-analysis

## Abstract

***Objectives:*** Cold hypersensitivity in the hands and feet (CHHF) and Raynaud's phenomenon (RP) are prevalent among Asian populations, especially among women, who exhibit a higher rate of cold hypersensitivity that may be associated with gynecological problems. In several countries, herbal medicine has effectively treated cold hypersensitivity symptoms. This systematic review and meta-analysis of the literature was undertaken to evaluate the efficacy of herbal medicine for the treatment of CHHF in adults.

***Design:*** Through March 31, 2018, comprehensive databases were searched, including MEDLINE, EMBASE, Cochrane Library, Chinese Academic Journal, and Japanese National Institute of Informatics, to identify relevant studies and extract data.

***Outcome measures:*** Primary: total effective rate (TER); secondary: skin temperature, peripheral blood flow, adverse events.

***Results:*** Fourteen randomized controlled trials (*n* = 974) were included. Thirteen studies with dichotomous values showed a significant reduction in CHHF and RP (risk ratio 0.31, 0.24–0.40) when comparing herbal medicine with/without Western medicine, and no treatment or Western medicine alone. Reductions in CHHF and RP were also observed between herbal medicine plus Western medicine and Western medicine alone (risk ratio 0.45, 0.24–0.86), as well as between herbal medicine and Western medicine alone (risk ratio 0.30, 0.21–0.41). In the only study using a placebo arm, herbal medicine was found to be superior to placebo in increasing skin temperature and peripheral blood flow. Six participants exhibited minor adverse drug reactions. Herbal medicine showed a superior TER, especially when combined with Western medicine, to Western medicine alone or placebo. However, there was a high risk of bias within all studies.

***Conclusion:*** Although herbal medicine shows potential to be a safe and effective treatment for CHHF and RP, the high risk of bias in all studies prevents definitive conclusions; thus, higher quality studies must be performed.

## Introduction

Cold hypersensitivity in the hands and feet (CHHF) is a common symptom that occurs in ∼20%–52% of the Eastern Asian population, particularly in women.^1,2^ Women have a higher risk of developing CHHF than men; notably, a Korean study researching twin genetics found that the ratio of women to men (who exhibit CHHF) is 3:2.^3^ Although the research and reviews related to CHHF have been increasing, the definition of CHHF has not been well established yet. In a previous study,^4^ the notion that “CHHF is a sensation of coldness in the hands and feet in an environment not considered cold by unaffected people or having a heightened cold sensation in a relatively low temperature area” or “answering ‘cold’ to both ‘are your hands cold or warm?’ and ‘are your feet cold or warm?’” has been accepted. CHHF is associated with Raynaud's phenomenon (RP), an episodic vasospastic disorder, as well as with gastric disorders^4^ and gynecological problems.^1,2^ Specifically, in women, CHHF has been associated with gynecological problems, such as infertility and dysmenorrhea.^5–10^ The pathophysiological mechanisms of CHHF or RP remain unclear.^11^

In conventional medicine, CHHF is the representative clinical symptom of RP; therefore, most patients with CHHF are prescribed antihypertensive drugs or vasodilators. However, in Eastern medicine, traditional Chinese medicine, traditional Korean medicine, and traditional Japanese medicine (Kampo medicine), coldness is viewed as the main pathogenic factor. In Yin–Yang theory, body temperature goes down as Yang energy is exhausted and Yin energy is replenished. Balancing the body's Yin and Yang energy with holistic and personalized approaches is the primary focus of CHHF treatment in Eastern and traditional medicine.

For more than 2000 years, herbal medicine has been used to regulate biological coldness in many countries; to the best of the authors' knowledge, no systematic review of the efficacy of herbal medicine for the treatment of CHHF has been conducted and published in English. Thus, a systematic review and meta-analysis of the literature were conducted to determine whether herbal medicine treatments are more effective in improving CHHF symptoms than other treatments or placebos.

## Materials and Methods

### Study design

The target participants were patients who exhibited either CHHF or primary RP, and who were receiving conventional medical treatment or no treatment. Patients with CHHF or primary RP were defined as those diagnosed mainly by their chief complaints, cold stress test, or thermography. CHHF is the manifestation of the symptoms, not a disease, so it has no unified code in the International Classification of Diseases.

Intervention was defined as herbal medicine treatments, either herbal medicine alone or herbal medicine plus Western medicine. Comparison was defined as no treatment, Western medicine, placebo, or herbal medicine.

### Data sources and search strategy

Studies were selected after a comprehensive search of the following databases: MEDLINE, EMBASE, CIHAHL, the Cochrane Library, China National Knowledge Infrastructure, Japanese National Institute of Informatics, and OASIS (Korea). The search spanned the time period from the inception of each database to March 31, 2018, and used specific search string combinations according to each database for subject limitations within the English, Chinese, Japanese, and Korean languages. Authors of the selected studies without raw data were contacted for the detailed raw data.

### Study selection and criteria

The target studies were randomized controlled trials that involved patients with CHHF or RP, if they met the following criteria: (1) randomized controlled trials that evaluated the effects of herbal medicine on patients with CHHF and (2) studies that provided the total effective rate (TER) or a visual analog scale (VAS) after completion of planned treatment. The exclusion criteria were as follows: (1) studies without a control group; (2) nonhuman clinical studies; (3) nonrandomized controlled trials; (4) studies that investigated the combined effects of herbal medicine and other therapies; (5) studies that did not provide any values (e.g., TER or VAS); and (6) studies that investigated CHHF caused by secondary RP, or by a specific disease (e.g., hypothyroidism, stroke, tumor, or diabetic neuropathy).

### Data extraction

Two evaluators independently extracted the data from the studies, and discrepancies were discussed and solved either by negotiation or by a third party. Primary outcomes included the TER, discomfort intensity (e.g., VAS), and a numerical rating scale; secondary outcomes included temperature differences in the hands and feet, peripheral blood flow, and adverse events. The experiment, control, population, and outcomes of each study are summarized in Table 1.

**Table 1.** Characteristics of the Included Studies

*No.*	*Study ID*	*Sample size (E/C)*	*Intervention (dose, frequency)*	*Comparison*	*Treatment course (days)*	*Outcome measurements*	*Adverse events*
1	Wang and Cai^13^	110 (55/55)	Dangguisinitang+Huangqiguizhiwuwutang (200 mL/day, bid)	Nifedipine 20 mg, bid	28	TER	Not reported
2	Guan and Li^14^	40 (20/20)	Tongmaitang (200 mL/day, bid)	Fufangdanshenpin	14	TER	Not reported
3	Xiao and Zheng^15^	40 (20/20)	Yanghetang (liquid dose per day unknown, bid)+Beraprost sodium 40 μg t.i.d.	Beraprost sodium 40 μg t.i.d.	60	TER	Not reported
4	Zhao and Yang^16^	66 (33/33)	Fumaxinguitang (liquid dose per day unknown, t.i.d.)+Calcium antagonist, adrenalin inhibitor, α-receptor antagonist	Calcium antagonist, adrenalin inhibitor, α-receptor antagonist	30	TER	Not reported
5	Zhang et al.^17^	50 (30/20)	Wenyangwuchongtongbitang (liquid dose per day and frequency unknown)	Metoprolol tartrate 50 mg bid	15	TER, nail capillary microcirculation	Not reported
6	Dou et al.^18^	53 (28/25)	Sinitang+Dangguisinitang (liquid dose per day unknown, bid)	Tolazoline 25 mg, Niacin 50 mg t.i.d.	20	TER	Not reported
7	Wu and Wang^19^	66 (42/24)	Tongmaijiejingsan (15 capsules [9 g of herb powder]/day, t.i.d.)	Tolazoline 25 mg, Niacin 50 mg t.i.d.	45	TER	Not reported
8	Park et al.^20^	80 (40/40)	Korean red ginseng Extract capsules (12 capsules [6 g of Korean red ginseng]/day, bid)	Placebo capsules	56	Skin temperature, CHHF severity, recovered temperature, temperature difference, HRV, SF36	Experimental group: itching (*n* = 1)
9	Nobuhiro et al.^21^	61 (31/30)	Gui Zhi Fu Ling Wan (extract granule 4.5 g [3 packs]/day, t.i.d.)+Vitamin E	Gui Zhi Fu Ling Wan	28	Symptoms, VAS, Lab, QOL, PoT	Experimental group: GOT increase (*n* = 1), Control group: GOT increase (*n* = 1), eczema (*n* = 1)
10	Shinji et al.^22^	58 (28/30)	Tokishigyakukagoshuyushokyoto (extract granule 7.5 g/day, t.i.d.)	Life style guidance	56	Blood flow, skin temperature	Experimental group: mild epigastric pain (*n* = 2)
11	Akiyama et al.^23^	44 (25/19)	Orengedokutou (extract granule 7.5 g/day, t.i.d.) Toukisyakuyakusan (extract granule 7.5 g/day, t.i.d.)+Sarpogrelate hydrochloride	Sarpogrelate hydrochloride	84	TER	Not reported
12	Ushiroyama et al.^24^	113 (58/55)	Wen-jing-tang (extract granule 7.5 g/day, t.i.d.)	Vitamin E	32	TER, blood flow	Not reported
13	Wang^25^	96 (48/48)	Dangguisinitang (liquid dose per day unknown, bid)	Nifedipine 20 mg, bid	28	TER, blood flow	Not reported
14	Dan^26^	96 (48/48)	Dangguisinitang (modified) (liquid dose per day unknown, t.i.d.)	Nifedipine 30 mg, t.i.d.	30	TER, nail capillary circulation	Not reported

C, control group; CHHF, cold hypersensitivity in the hands and feet; E, experimental group; GOT, glutamic oxaloacetic transaminase; HRV, heart rate variability; PoT, period of time of recovering from coldness; QOL, quality of life; SF36, Short form 36; TER, total effective rate; VAS, visual analog scale.

### Study quality assessment

Two reviewers independently evaluated the study's methodological quality using the Cochrane Handbook for Systematic Review of Intervention Version 5.1.0. A quality assessment included the following seven items: random sequence generation (selection bias), allocation concealment (selection bias), blinding of participants and personnel (performance bias), blinding of outcome assessor (detection bias), incomplete outcome data (attrition bias), selective reporting (reporting bias), and other biases. Each assessment was categorized into three levels: low risk, unclear risk, and high risk. Then, the trials were categorized as having high risk of bias (at least one item had a high risk of bias), unclear risk of bias (at least one item had an unclear risk of bias), or low risk of bias (all the items had a low risk of bias).

### Data synthesis and analysis

For conducting the meta-analysis, statistical heterogeneity among the included studies was assessed using the *I*^2^ index before the meta-analysis.^12^ If the *I*^2^ statistic was <50%, the heterogeneity was regarded as acceptable, and the fixed-effects model was adopted. If it was >50%, the random-effects model was used, because statistical heterogeneity would be significant. The risk ratio was used for dichotomous outcomes, and the mean difference was used for continuous outcomes as effect estimates. RevMan5.3 (The Cochrane Collaboration, Oxford, United Kingdom) was used for meta-analysis. A funnel plot to check for the existence of publication bias might be performed, but in this study the number of included studies was small, and therefore, it was not executed.

### Outcome measurements

The outcome was TER or VAS for symptom severity or temperature differences. The TER was evaluated using four items such as much improved, improved, not changed, and exacerbated. Much improved and improved were regarded as effective. The primary outcome comprised the TER or VAS; secondary outcomes were skin temperature, peripheral blood flow, and adverse events.

## Results

### Study search results

**FIG. 1.**
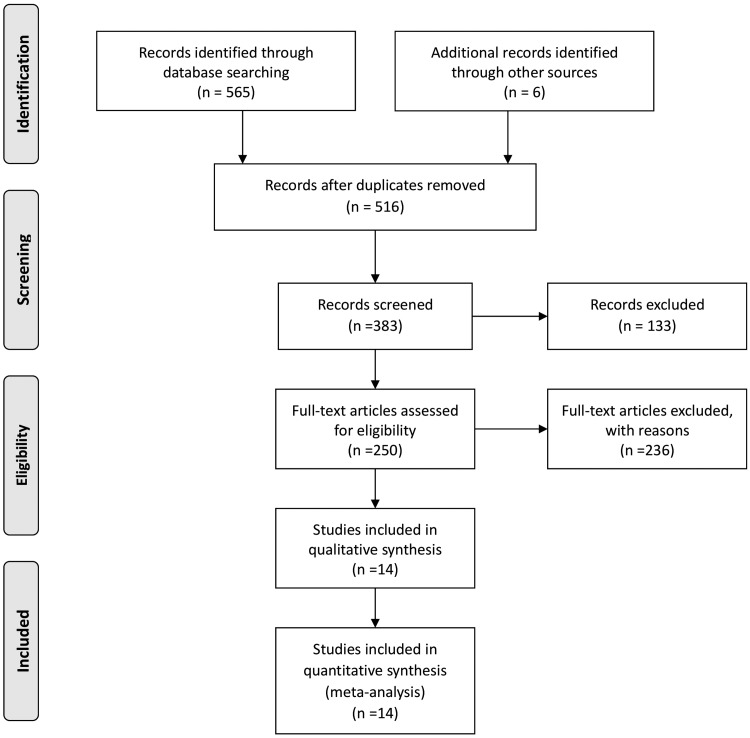
PRISMA chart for cold hypersensitivity in the hands and feet.

Through electronic searching, 571 records were identified. After the removal of duplicates (*n* = 55), 516 records were screened. Initially, 383 studies met the inclusion criteria, and these studies then underwent full-text screening. In total, 14 randomized controlled trials were included.^13–24^ The study selection process is illustrated in Figure 1.

### Characteristics of the included studies

A total of 974 participants in 14 trials, with sample sizes ranging from 19 to 58 subjects, were included in the analysis. Nine of the trials^13–19,25,26^ were conducted in China, one trial^20^ was conducted in Korea, and four trials^21–24^ were conducted in Japan. Seven of the trials^13,17–19,24–26^ compared herbal medicine with standard Western medications. Three of the trials^15,16,23^ compared herbal medicine plus Western medications with Western medications. Two of the trials^20,22^ compared herbal medicine with placebo or no treatment. One of the trials^21^ compared herbal medicine plus Western medication with herbal medicine. One of the trials^14^ compared herbal medicine with a herbal medicine preparation (Fufangdanshenpin). The standard Western medications included nifedipine, beraprost sodium, adrenalin inhibitor, metoprolol tartrate, tolazoline, and sarpogrelate hydrochloride. The TER was measured in nine trials,^13–19,23–26^ skin temperature was measured in two trials,^20,22^ and peripheral blood flow by laser Doppler fluxmeter was measured in two trials.^22,24–26^ Herbal medicine treatment administration ranged from 2 to 12 weeks.

### Quality assessment of the included trials

All the included trials mentioned randomization, but only seven of the trials^13,14,19,20,22,25,26^ described the method of randomization, including the random number table or randomization program. Only two trials^20,22^ mentioned a process for allocation concealment by an independent statistician through the end of the study. There was no information related to blind outcome assessments; therefore, all the trials were considered to have a high risk of bias.

### Outcomes of interventions

#### Primary outcomes

##### Total effective rate

**FIG. 2.**
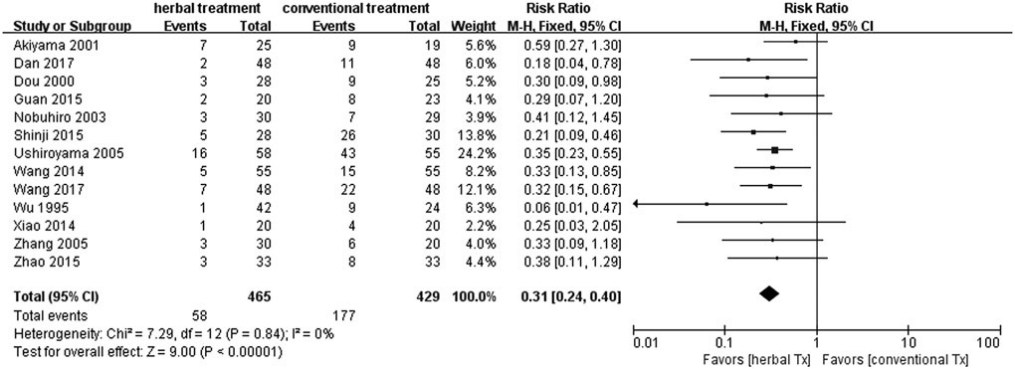
Forest plot and risk of bias in the effect of herbal medicine treatment versus placebo or standard Western medications for CHHF using the fixed-effects model. CHHF, cold hypersensitivity in the hands and feet.

**FIG. 3.**
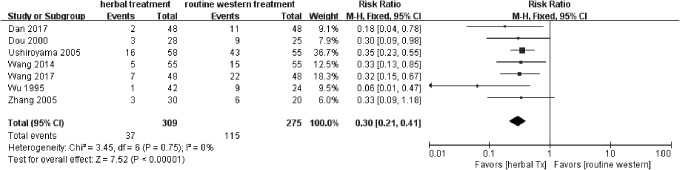
Forest plot and risk of bias in the effect of herbal medicine treatment versus standard Western medications for CHHF using the fixed-effects model. CHHF, cold hypersensitivity in the hands and feet.

**FIG. 4.**
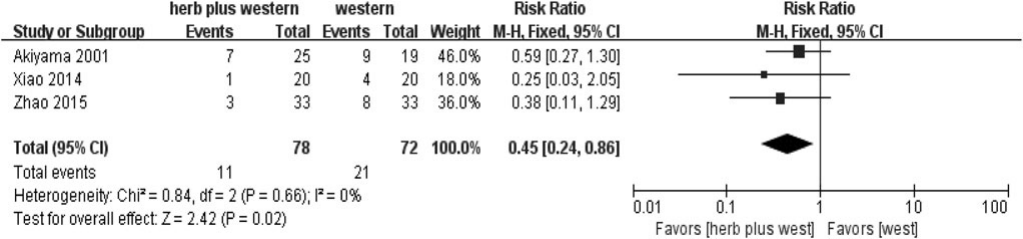
Forest plot and risk of bias in herbal medicine treatment plus standard Western medications versus standard Western medications for CHHF using the fixed-effects model. CHHF, cold hypersensitivity in the hands and feet.

Thirteen trials^13–19,21,22,25,26^ that measured the TER or VAS showed a significant difference in favor of herbal medicine with/without Western medicine compared with no treatment or Western medicine (risk ratio 0.31). Eleven trials^13–19,23–26^ measured TER. All nine Chinese studies and two of the Japanese studies included TER (Fig. 2). Seven trials^13,17–19,24–26^ compared herbal medicine treatment with standard Western medications and revealed a significant difference in favor of herbal medicine (risk ratio 0.30; Fig. 3); three trials^15,16,23^ compared herbal medicine treatment plus standard Western medications with standard Western medications alone. They also showed a significant difference in favor of herbal medicine plus standard Western medicine (risk ratio 0.45; Fig. 4). One trial^14^ compared herbal medicine with a herbal medicine preparation (Fufangdanshenpin).

#### Secondary outcomes

##### Skin temperature

**FIG. 5.**
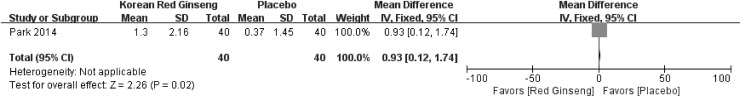
Forest plot and risk of bias in herbal medicine treatment (Korean red ginseng) versus placebo for CHHF using the fixed-effects model. CHHF, cold hypersensitivity in the hands and feet.

Two trials^20,22^ evaluated outcomes using skin temperature. One trial^22^ detected no significance between the two forms of treatment, whereas the other trial^20^ indicated that Korean red ginseng was more effective than placebo in reducing skin temperature differences between the palms and upper arms (mean difference = 0.93, 95% confidence interval = 0.12–1.74, *p* = 0.02; Fig. 5).

### Peripheral blood flow

Four trials^22,24–26^ mentioned evaluating outcomes based on peripheral blood flow using a laser Doppler fluxmeter. First trial^22^ showed that tokishigyakukagoshuyushokyoto (TJ-38) was better than the control (life style guidance) for recovering blood flow 10 min after a 30-s cold water test (*p* = 0.007). The second trial^24^ showed that wen-jing-tang (unkei-to) was better than the control (vitamin E administered) for increasing the peripheral surface blood flow of the tiptoe (*p* = 0.0068). Third trial^25^ showed that Danggui Sini Tang was better than Western medicine for changing the finger arterial blood flow peak in diastolic period. Fourth trial^26^ showed that Danggui Sini Tang was better than Western medicine for improving nail capillary blood flow.

### Adverse events

One trial^21^ reported that a participant in the experimental group (Guizhi fuling wang+vitamin E) and two participants in the control group (guizhi fuling wan) had adverse drug reactions of glutamic oxaloacetic transaminase increase and eczema. One trial^20^ reported that one participant in the experimental group (Korean red ginseng) presented with slight itching that disappeared after a few days. One trial^22^ reported that two participants in the experimental group (TJ-38) presented with a light stomachache. For these participants, the medication dosage was reduced by half, and the symptoms disappeared. The other nine trials^13–19,23,24^ did not mention any adverse drug reactions, and there were no serious adverse drug reactions reported in the 14 trials.

## Discussion

**FIG. 6.**
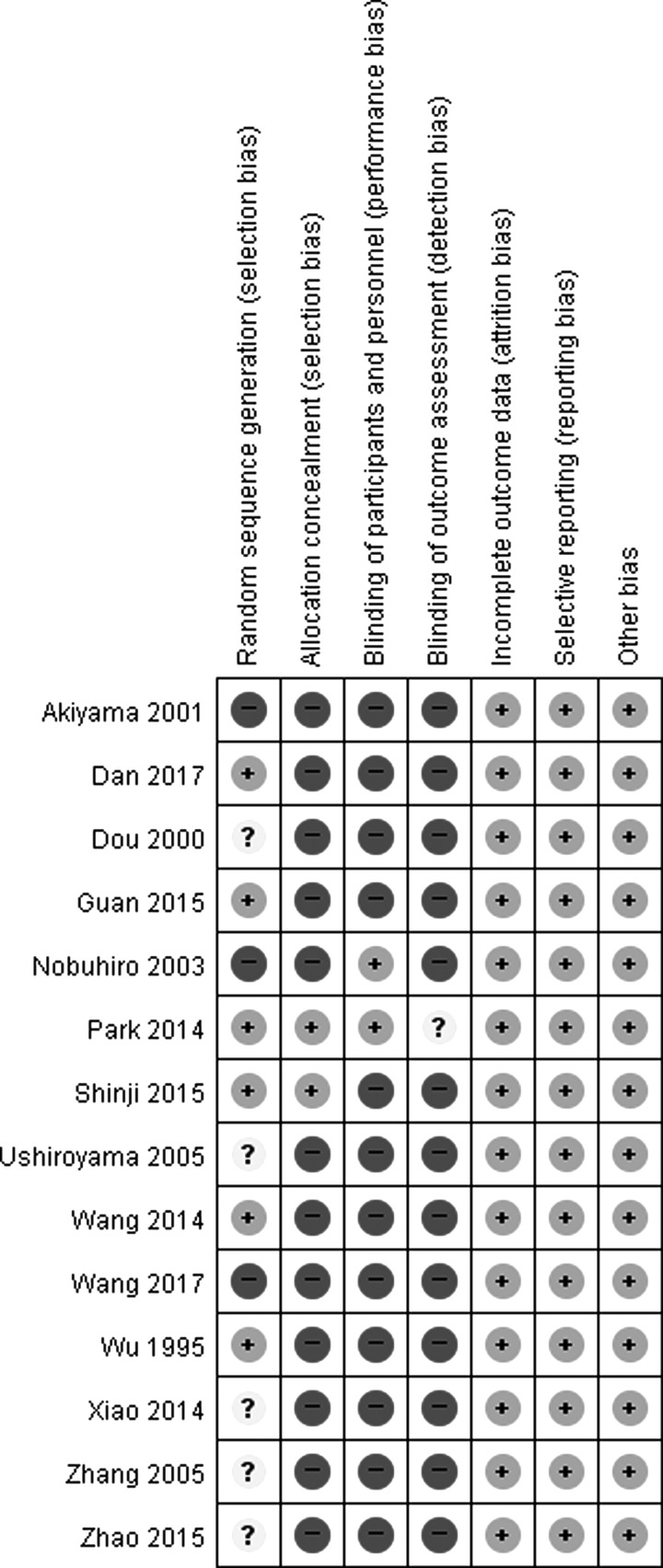
Risk of bias assessment in the included studies.

A total of 14 studies (974 CHHF patients) were included in this review. From the results of the meta-analysis on 13 of the studies^13–19,21–26^ that had dichotomous values in terms of TER, a significant difference was observed between the herbal medicine group and the control group (relative risk 0.31 [0.24–0.40]; Fig. 2). The findings between the herbal medicine group and the Western medicine group^13,17–20,24–26^ were similar in all 13 studies^13–19,21–26^ (relative risk 0.30 [0.21–0.41]; Fig. 3). When comparing herbal medicine plus Western medicine with Western medicine alone,^15,16,23^ the results showed a significant difference (relative risk 0.45 [0.24–0.86]; Fig. 4). Consequently, although the results provide preliminary evidence that herbal medicine is a relatively safe and effective treatment for CHHF and RP, the high risk of bias in the existing studies makes ours a cautious conclusion. A significant improvement was observed in favor of the herbal medicine group in TER. One trial^20^ showed that Korean red ginseng has some therapeutic value for improving CHHF (Fig. 5), which had a high evidence level, but one of the participants taking the Korean red ginseng experienced itching as an adverse event. The overall quality of the 13 included studies suggested that there were considerable methodological problems of selection bias, performance bias, and detection bias (Fig. 6).^13–19,21–26^ All of the studies were small-population studies without sample size calculations. Nearly two-thirds of the included studies did not report on adverse events.^13–19,23–26^ Consequently, herbal medicine could be a safe and effective treatment for CHHF and RP, but the current evidence remains sparse due to risk of bias, requiring further studies on the subject.

Herbal preparations are shown in Table 2, which summarizes the contents of the herbal medicines according to dose per day. Analysis of the contents of the herbal medicines revealed that the most common herbal medicines were *Cinnamomi ramulus* or *Cinnamomi cortex* and *Zingiberis rhizoma*. Cinnamomi was present in 12 of 14 articles, and Zingiberis was present in 7 of 14 articles. These two herbal medicines have warm properties and a pungent smell in common.

**Table 2.** Herbal Composition and Preparations

*No.*	*Study ID*	*Name of herbal preparation*	*Composition (dose per day) (unit: g)*	*Preparation type*
1	Wang and Cai^13^	Dangguisinitang+Huangqiguizhiwuwutang	*Astragali radix* 50, *Cinnamomi ramulus* 20, *Angelicae gigantis radix* 20, *Salvia miltiorrhiza Bunge* 20, *Paeoniae radix alba* 20, *Persicae semen* 10, *Bupleuri radix* 10, *Ponciri fructus immaturus* 10, *Aconiti lateralis radix Preparata* 10, *Zingiberis rhizoma* 10	Liquid type after water boiling
2	Guan and Li^14^	Tongmaitang	*Aconiti tuber* 50, *Aconiti Kusnezoffii tuber* 50, *Clematidis radix* 30, *Artemisiae Argyi herba* 25, *Lycopodii herba* 25, *Cinnamomi ramulus* 15	Liquid type after water boiling
3	Xiao and Zheng^15^	Yanghetang	*Rehmanniae radix preparata* 30, *Cervi Cornus Colla* 20, *Zingiberis rhizoma* 12, *Cinnamomi cortex Spissus* 15, *Angelicae gigantis radix* 12, *Spatholobi caulis* 12, *Ephedrae herba* 4, *Sinapis semen* 9, *Zaocys* 12, *Lumbricus* 9, *Glycyrrhizae radix et rhizoma* 3	Liquid type after water boiling
4	Zhao and Yang^16^	Fumaxinguitang+Calcium antagonist, adrenalin inhibitor, α-receptor antagonist	*Aconiti Koreani tuber* 30, *Ephedrae herba* 9, *Asiasari radix* 5, *Cinnamomi ramulus* 12, *Cinnamomi Cortex Spissus* 20, *Zingiberis rhizoma* 15, *Astragali radix* 20, *Carthami flos* 10, *Salviae miltiorrhizae radix* 20, *Angelicae gigantis radix* 10, *Paeoniae radix* r*ubra* 15, *Glycyrrhizae radix et rhizoma* 5(t.i.d.)	Liquid type after water boiling
5	Zhang et al.^17^	Wenyangwuchongtongbitang	*Rehmanniae radix Preparata* 30, *Cinnamomi cortex Spissus* 3, *Ephedrae herba* 6, *Sinapis smen* 9, *Zingiberis rhizoma* 6, *Cinnamomi ramulus* 9, *Paeoniae radix alba* 9, *Salviae miltiorrhizae radix* 30, *Angelicae gigantis radix* 15, *Cnidii rhizoma* 15, *Cervi Cornus Colla* 9, *Astragali radix* 30, *Scorpion* 5, *Scoopendrae corpus* 3, *Eupolyphaga sinensis Walker* 6, *Hirudo* 3, *Cicadidae periostracum* 6, *Glycyrrhizae radix et rhizoma* 6	Liquid type after water boiling
6	Dou et al.^18^	Sinitang+Dangguisinitang	*Aconiti Lateralis radix preparata* 10, *Zingiberis rhizoma* 15, *Glycyrrhizae radix et rhizoma* 15, *Cinnamomi ramulus* 15, *Paeoniae radix rubra* 15, *Astragali radix* 15, *Corydalis tuber* 15, *Angelicae gigantis radix* 20, *Asiasari radix* 5, *Salviae miltiorrhizae radix* 25	Liquid type after water boiling
7	Wu and Wang^19^	Tongmaijiejingsan	*Ephedrae herba* 20, *Osterici radix* 20, *Angelicae gigantis radix* 30, *Bupleuri radix* 30, *Cinnamomi ramulus* 30, *Paeoniae radix alba* 40, *Asiasari radix* 15, *Akebiae caulis* 15, *Salviae miltiorrhizae radix* 50, *Eupolyphaga sinensis Walker* 10, *Phrymaceae herba* 25	After making herb medicine into powder, fill powder into capsules (each capsule has 0.6 g of herb powder)
8	Park et al.^20^	Korean red ginseng Extract capsules	Korean red ginseng capsules	Extract capsules
9	Nobuhiro et al.^21^	Gui Zhi Fu Ling Wan	Extract granule 4.5 g (3 packs)/day [Gui Zhi Fu Ling Wan Extract 600 mg, *Cinnamon bark* 450 mg, *Moutan Cortex radicis* 450 mg, *Persicae semen* 450 mg, *Hoelen* 450 mg, *Paeoniae radix* 450 mg]	Extract granule
10	Shinji et al.^22^	Tokishigyakukagoshuyushokyoto	Extract granule 7.5 g/day, t.i.d. *Zizyphi fructus* 5, *Cinnamomi cortex* 3, *Paeoniae radix* 3, *Angelicae radix* 3, *Akebiae caulis* 3, *Glycyrrhizae radix* 2, *Evodiae fructus* 2, *Asiasari radix* 2, *Zingiberis rhizoma* 1	Extract granule
11	Akiyama et al.^23^	Orengedokutou or Toukisyakuyakusan	Orengedokutou (extract granule 7.5 g/day, t.i.d.)	Extract granule
			*Scutellariae radix* 3, *Coptidis rhizoma* 2, *Phellodendri cortex* 1.5, *Gardeniae fructus* 2	
			Toukisyakuyakusan (extract granule 7.5 g/day, t.i.d.)	
			*Paeoniae radix* 4, *Atractylodis rhizoma alba* 4, *Alismatis rhizoma* 4, *Hoelen* 4, *Cnidii rhizoma* 3, *Angelicae radix* 3	
12	Ushiroyama et al.^24^	Wen-jing-tang	Extract granule 7.5 g/day, t.i.d.	Extract granule
			*Cinnamomi cortex* 2, *Evodiae fructus* 1, *Angelicae radix* 33, *Cnidium rhizoma* 2, *Paeoniae radix* 2, *Moutan cortex* 2, *Ophiopogonis tuber* 4, *Asini Corii Collas* 2, *Ginseng radix* 2, *Glycyrrhizae radix* 2, *Zingiberis rhizoma* 1, *Pinelliae tuber* 4	
13	Wang^25^	Dangguisinitang	*Astragali radix* 15, *Angelicae gigantis radix* 12, *Paeoniae radix alba* 12, *Mori ramulus* 12, *Cinnamomi ramulus* 10, *Tetrapanacis medulla* 10, *Zaocys dhumnades* 10, *Spatholobi caulis* 10, *Zizyphi fructus* 10, *Asiasari radix* 3, *Glycyrrhiza uralensis Fischer* 5	Liquid type after water boiling
14	Dan^26^	Dangguisinitang (modified)	*Cinnamomi ramulus* 15, *Astragali radix* 30, *Angelicae gigantis radix* 30, *Zaocys dhumnades* 15, *Allolobophora trapezoids* 15, *Asiasari radix* 3, *Akebiae caulis* 10, *Paeoniae radix alba* 20, *Glycyrrhiza uralensis Fischer* 9, *Zizyphi fructus* 10 pieces, permit to add some herbal medicines according to the patient's symptom	Liquid type after water boiling

*Cinnamomi ramulus*, or *Cinnamomi cortex*, being one of the most important spices worldwide, was traditionally administered for colds, flu, and digestive problems, and is still used in much the same way today. The key constituents are cinnamaldehyde, eugenol, tannins, coumarins, and mucilage. In both India and Europe, as well as East Asia, Cinnamomi has been traditionally taken as a warming herb for “cold” conditions, often in combination with Zingiberis. The herb stimulates the circulation, especially to the fingers and toes. It is also a traditional remedy for digestive problems, such as nausea, vomiting, and diarrhea, as well as for aching muscles and other symptoms of viral conditions, such as colds.^27^

Zingiberis is well researched; its key constituents are zingiberene, gingerol, and shogaols. Zingiberis was traditionally and is currently used to treat digestive problems, to serve as a circulatory stimulant, and to combat respiratory conditions. In traditional Eastern medicine, fresh Zingiberis is administered for fever, headache, and aching muscles, whereas dried Zingiberis is used for “internal cold” with symptoms such as cold hands, a weak pulse, and a pale complexion.^27^

There are strong points in this review. Primarily, to the authors' knowledge, this is the first review on the effects of herbal medicines used in the management of CHHF or RP. Herbal medicine is an important intervention in East Asian medicine, so the clinical evidence needed to be assessed. From this review, the authors tried to evaluate the clinical evidence, as well as the clinical application of herbal medicine. Second, an extensive search strategy was adopted without imposing any language limitations, including Chinese, English, Korean, and Japanese. Half of the included studies in this review were conducted in China and written in Chinese, which suggests that the strategy for the inclusion of the studies was appropriate. In this regard, studies of CHHF and RP were prevalent in East Asian countries, along with a high prevalence rate of CHHF. Unfortunately, a clear cause of this phenomenon has not been elucidated. It was assumed that the East Asian countries with high prevalence rates are located in the temperate climate region, and on islands or peninsulas. Third, the intervention details were summarized, including the herbal substance names provided in the prescriptions, the herbal medicine composition, the preparation type, and the dose frequency, and duration (Table 2). This information helps practitioners to apply these interventions in their own clinical practice.

This review also has limitations: The most important limitation originates from the included studies themselves, which are prone to serious risk of bias. Nearly two-thirds of the studies did not report on adverse events. In addition, most of the trials were published in China, which may indicate publication bias. Therefore, these risks of bias may impact the evidence to some extent. Second, the authors could not confirm whether all the included studies were really randomized controlled trials, because there is a report that found many clinical trials conducted in China could not be included in the authentic criteria of randomized controlled trials.^28,29^ A third limitation of the study is that dosage of the herbal intervention was not reported in 6 of 14 studies, and therefore those trials cannot be replicated.

On the basis of these results and in the near future, a randomized controlled trial of the herbal medicine extract was planned, using a double-blind method, to treat CHHF.

## Conclusion

Although herbal medicine shows potential to serve as a safe and effective treatment for CHHF and RP, the high risk of bias in all studies prevents definitive conclusions; thus, higher quality studies must be performed.
